# Phase III pilot study of dose escalation using conformal radiotherapy in prostate cancer: PSA control and side effects

**DOI:** 10.1038/sj.bjc.6602301

**Published:** 2005-02-01

**Authors:** D P Dearnaley, E Hall, D Lawrence, R A Huddart, R Eeles, C M Nutting, J Gadd, A Warrington, M Bidmead, A Horwich

**Affiliations:** 1 Academic Department of Radiotherapy & Oncology, Institute of Cancer Research, Sutton, Surrey SM2 5PT, UK; 2 Clinical Trials & Statistics Unit (ICR-CTSU), Institute of Cancer Research, Sutton, Surrey SM2 5PT, UK; 3 The Royal Marsden NHS Foundation Trust, Sutton, Surrey SM2 5PT, UK

**Keywords:** prostate cancer, phase III trial, conformal radiotherapy, dose escalation, radiation toxicity, PSA

## Abstract

Radical radiotherapy is a standard form of management of localised prostate cancer. Conformal treatment planning spares adjacent normal tissues reducing treatment-related side effects and may permit safe dose escalation. We have tested the effects on tumour control and side effects of escalating radiotherapy dose and investigated the appropriate target volume margin. After an initial 3–6 month period of androgen suppression, 126 men were randomised and treated with radiotherapy using a 2 by 2 factorial trial design. The initial radiotherapy tumour target volume included the prostate and base of seminal vesicles (SV) or complete SV depending on SV involvement risk. Treatments were randomised to deliver a dose of 64 Gy with either a 1.0 or 1.5 cm margin around the tumour volume (1.0 and 1.5 cm margin groups) and also to treat either with or without a 10 Gy boost to the prostate alone with no additional margin (64 and 74 Gy groups). Tumour control was monitored by prostate-specific antigen (PSA) testing and clinical examination with additional tests as appropriate. Acute and late side effects of treatment were measured using the Radiation Treatment and Oncology Groups (RTOG) and LENT SOM systems. The results showed that freedom from PSA failure was higher in the 74 Gy group compared to the 64 Gy group, but this did not reach conventional levels of statistical significance with 5-year actuarial control rates of 71% (95% CI 58–81%) in the 74 Gy group *vs* 59% (95% CI 45–70%) in the 64 Gy group. There were 23 failures in the 74Gy group and 33 in the 64 Gy group (Hazard ratio 0.64, 95% CI 0.38–1.10, *P*=0.10). No difference in disease control was seen between the 1.0 and 1.5 cm margin groups (5-year actuarial control rates 67%, 95% CI 53–77% *vs* 63%, 95% CI 50–74%) with 28 events in each group (Hazard ratio 0.97, 95% CI 0.50–1.86, *P*=0.94). Acute side effects were generally mild and 18 weeks after treatment, only four and five of the 126 men had persistent ⩾Grade 1 bowel or bladder side effects, respectively. Statistically significant increases in acute bladder side effects were seen after treatment in the men receiving 74 Gy (*P*=0.006), and increases in both acute bowel side effects during treatment (*P*=0.05) and acute bladder sequelae (*P*=0.002) were recorded for men in the 1.5 cm margin group. While statistically significant, these differences were of short duration and of doubtful clinical importance. Late bowel side effects (RTOG⩾2) were seen more commonly in the 74 Gy and 1.5 cm margin groups (*P*=0.02 and *P*=0.05, respectively) in the first 2 years after randomisation. Similar results were found using the LENT SOM assessments. No significant differences in late bladder side effects were seen between the randomised groups using the RTOG scoring system. Using the LENT SOM instrument, a higher proportion of men treated in the 74 Gy group had Grade ⩾3 urinary frequency at 6 and 12 months. Compared to baseline scores, bladder symptoms improved after 6 months or more follow-up in all groups. Sexual function deteriorated after treatment with the number of men reporting some sexual dysfunction (Grade⩾1) increasing from 38% at baseline to 66% at 6 months and 1 year and 81% by year 5. However, no consistent differences were seen between the randomised groups. In conclusion, dose escalation from 64 to 74 Gy using conformal radiotherapy may improve long-term PSA control, but a treatment margin of 1.5 cm is unnecessary and is associated with increased acute bowel and bladder reactions and more late rectal side effects. Data from this randomised pilot study informed the Data Monitoring Committee of the Medical Research Council RT 01 Trial and the two studies will be combined in subsequent meta-analysis.

Radiotherapy is one of the curative treatment options for localised prostate cancer (Consensus conference, 1987; COIN Guidelines, 1999). Considerable advances in radiation technology over the last decade have led to the development of conformal radiation treatments, which more closely match the high dose volume to the tumour target while reducing the radiation to dose-limiting normal tissues (Fuks and Horwich, 1993). The potential advantage of these techniques is to enable a reduction in radiation-related side effects as well as permitting the safe delivery of high doses of radiation, which might improve treatment efficacy. Institutional experiences and results from phase I/II studies suggest that both these goals may be achievable (Sandler *et al*, 1992; Hanks *et al*, 1998; Zelefsky *et al*, 1998) and that dose/response relationships exist for tumour control as well as dose/volume/complication relationships for the development of late normal tissue damage. However, only two phase III randomised trials using photon beam treatment have been reported. In the first, we compared conventional and conformal radiotherapy (CFRT) at a standard dose of 64 Gy (Dearnaley *et al*, 1999) and showed a significant reduction in the dose-limiting late side effect of proctitis using CFRT. In the second trial, conventional radiotherapy (70 Gy) was compared with a mixed schedule of conventional and CFRT to a dose of 78 Gy. In this study, an improvement in failure-free survival with higher dose was suggested but radiation proctitis was also increased (Pollack *et al*, 2000).

An alternative strategy to improve the local treatment results of radiation therapy is to use an initial period of androgen suppression/blockade. Potential advantages of combined modality treatment include an additive or synergistic effect on tumour cell kill and also a reduction in radiation target volume (Dearnaley, 2001). Four phase III randomised trials have reported benefits in tumour control compared to radiation alone (Laverdiere *et al*, 1997, 2004; Porter *et al*, 1998; Pilepich *et al*, 2001), and an overview of the Radiation Treatment and Oncology Groups (RTOG), experience suggested an overall improvement in survival using neoadjuvant androgen suppression in addition to radiotherapy (Roach III *et al*, 2000). Several groups have measured the reduction in prostate and prostate target volume after initial hormone treatment, which varied between 25 and 41% and showed a complimentary increase in the sparing of rectum and bladder when initial hormone treatment was combined with CFRT (Zelefsky *et al*, 1994; Forman *et al*, 1995; Dearnaley, 2000). However, there is currently no evidence to suggest whether or not the initial or posthormone treatment prostate volume should be used to construct the radiation target volume. This issue is complicated by the need to define a ‘safety margin’ around target tissues to account for the day to day variations in patient and prostate position and the accuracy of radiation delivery (Tinger *et al*, 1998; Wu *et al*, 2001).

In our study we wanted to evaluate the role of dose escalation using conformal radiotherapy in conjunction with initial androgen suppression. We constructed a phase III randomised trial using a 2 × 2 factorial design to assess, firstly, our standard dose of 64 Gy compared to the escalated dose of 74 Gy and, secondly, to compare a radiation ‘safety margin’ of 1.0 cm to that of 1.5 cm. The trial was initially designed as a single institution study at the Royal Marsden NHS Trust (RMT) and Institute of Cancer Research (ICR), but the opportunity came to develop the protocol further with the then newly formed Medical Research Council (MRC) Radiotherapy Working Party. A national multicentre trial (MRC RT01) commenced in January 1998 (Seddon *et al*, 2000; Sydes *et al*, 2004) at which time recruitment to our single institutional trial stopped. We had previously agreed with the MRC RT01 trial coordinating committee that the single institution trial would act as a ‘pilot study’ for the national trial, all data being sent to the MRC Data Monitoring Committee, and that in due course the trials would be reported separately before being combined in meta-analysis. Dose-limiting late side effects of radiation treatment may take 2 years or more to develop after therapy, and this pilot study allowed the national study to proceed with an additional degree of safety.

## PATIENTS AND METHODS

### Eligibility

The study was approved by the RMT and ICR Clinical Research and Ethics Committees. All men participating in the trial gave written informed consent. Patients with histologically proven T1b-T3b N0 M0 (UICC, 1997) adenocarcinoma of the prostate were eligible provided there was no past or current medical history, which made radical radiotherapy inappropriate, and there had been no previous androgen suppression or pelvic radiotherapy. Patients were not excluded on the basis of pretreatment prostate-specific antigen (PSA) levels alone and there was no specified upper age limit to trial entry.

### Pretreatment investigations

All men were assessed by clinical history and physical examination including digital rectal examination (DRE). Full blood count and biochemistry including creatinine, alkaline phosphatase and PSA levels were measured. Prostate-specific antigen samples were taken at least 10 days after any biopsy procedure and before rectal examination. Histopathology was reviewed at the RMT, using either Gleason or WHO reporting systems (Murphy *et al*, 1994). Staging of the primary disease was by DRE, supplemented by transrectal ultrasound (TRUS) and magnetic resonance imaging (MRI). Clinical staging was used to assign risk-group categories unless there was considered to be unequivocal evidence of upstaging on MRI. All patients had lymph node assessment using computer tomography (CT) or MRI. All patients had a bone scan with any appropriate correlative X-rays.

## TREATMENT

### Neoadjuvant androgen suppression

Androgen suppression was achieved using monthly depot injections of a luteinising hormone-releasing hormone analogue (LHRHa) using cyproterone acetate (CPA) 100 mg three times daily to prevent testosterone ‘flare’. Cyproteronone acetate was commenced 1 week prior to the first LHRHa and discontinued after a further 2 weeks. Luteinising hormone-releasing hormone analogue treatment was given for 3–6 months before radiotherapy with testosterone suppression continuing until the end of radiotherapy, after which all treatment was discontinued.

### Radiotherapy treatment

Radiotherapy treatment was designed on planning CT scans using a slice interval of 5 mm, which were performed approximately 12 weeks after commencement of hormone therapy. Patients were treated supine, with a comfortably full bladder. No contrast agents were given. Positioning was achieved using laser alignment of anterior and lateral tattoos sited in the plane of the superior border of the symphysis pubis. Ankle stops were used to aid immobilisation (Nutting *et al*, 2000). Outlining of target and normal tissues (rectum, bladder, femoral heads) was carried out on contiguous CT slices; the rectum and bladder were outlined as ‘solid’ organs using IGE Target or Cadplan systems. The bladder was contoured from apex to dome and rectum from the anus (at the level of the ischial tuberosities) for 14 cm or to the point at which the rectosigmoid junction was identified. Volumes of target tissues (prostate +/− seminal vesicles) were defined as in Table 1 according to ICRU Report 50 (ICRU, 1993). Patients were stratified into low- or moderate-risk groups for seminal vesicle involvement (Roach III, 1993). Patients with a risk of seminal vesicle involvement of <15% had the gross tumour volume defined as prostate and base of seminal vesicles alone; those patients with a higher risk of involvement had the seminal vesicles included in the phase I gross tumour volume (GTV1). All patients randomised to receive 74 Gy had a boost to the prostate only (GTV2). Patients were additionally randomised to have either a 0.5 or 1.0 cm margin added to the GTV1 to create the clinical target volume (CTV1). A further 0.5 cm was added to form the planning target volume (PTV1). The phase II boost treatments were given to the prostate only with no added margin (GTV2=CTV2=PTV2). The rationale for the randomisation between a 0.5 and 1.0 cm margin between the GTV1 and CTV1 was based on our previous experience using neoadjuvant androgen suppression (Dearnaley, 2000). We observed an approximate 50% reduction in prostate volume from a mean of 84 to 47 cm^3^. Assuming a spherical shape, this corresponds to a change in radius of 0.5 cm (2.7 cm reducing to 2.2 cm). All patients received initial treatment to PTV1 to a dose of 64 Gy in 2 Gy fractions treating daily over a period of 6 1/2 weeks. Patients randomised to the escalated dose arm received an extra five fractions of 2 Gy given over 1 week to the PTV2. Doses were defined at the isocentre. A three-field plan (anterior and left/right lateral or posterior oblique fields) was used for phase I plan (Khoo *et al*, 2000) with a six-field arrangement (left and right, anterior/posterior oblique and lateral fields) for phase 2 (Pickett *et al*, 1994). Treatments were delivered using 6–10 MV photons and fields shaped using multileaf collimators (MLC) or customized, shaped blocks. Orthogonal simulator films were taken to verify the orientation and alignment of the planned fields. Port films/images were taken initially on a daily basis and subsequently on a weekly basis to verify treatment accuracy.

### Patient follow-up

Acute bowel and urinary side effects were assessed weekly during therapy (weeks 1–6) and week 8, 10 and 18 using the RTOG system (Cox *et al*, 1995). Late radiation side effects were assessed using RTOG criteria (Lawton *et al*, 1991) and also the RTOG/EORTC LENT SOM (1995) (Late Effects Normal Tissue – Subjective Objective Management) classification at 6, 12, 18 and 24 months after treatment and thereafter annually. The RTOG system gives a consolidated score of all bladder or bowel symptoms on a six-point scale. The majority of patients will develop any signs of late morbidity within the first 2 years of treatment. The LENT SOM questionnaire comprises 13 questions on rectal function, 14 on bladder function, seven on sexual dysfunction and data were also collected on potential small intestine/colon, skin/subcutaneous tissue and bone side effects. Each question is answered on a five-point scale. The highest score from each set of questions has been reported when summarising results. Assessment of disease status was made using PSA measurements, clinical assessment and DRE at 6 weekly intervals during hormone therapy, then at 3, 6, 12, 18 and 24 months and then at annual intervals. Prostate-specific antigen was measured using the Hybritech enzyme immunoassay and the Roche immunometric assay prior to 1997. These assays provided results to the nearest ng ml^−1^ with a lower limited detection of 1 ng ml^−1^. In January 1997, the Abbot AXSYM assay was adopted with a lower limit of 0.1 ng ml^−1^. Given the limitations of the assays used during the earlier years of the study, we defined biochemical failure as either two consecutive rises in PSA ⩾2 ng ml^−1^, or the recommencement of androgen deprivation. The date of PSA failure was taken as the date of the first PSA value ⩾2 ng ml^−1^, or the date of starting androgen deprivation, respectively.

### Trial design, end points and statistics

The trial was designed as a prospective randomised phase III study using a 2 × 2 factorial design to study dose escalation and treatment volume. Patients' radiotherapy treatments were randomised between (a) total dose of 74 *vs* 64 Gy; (b) treatment margin (GTV-PTV) of 1.0 *vs* 1.5 cm.

Comparisons have been made between the randomised groups only. Independent randomisation was undertaken by ICR Clinical Trials and Statistics Unit using a randomised permuted block design. Stratification of patients was according to calculated risks of seminal vesicle involvement (Roach III, 1993). In a trial of this type, it was impractical to use any ‘blinding’ procedures.

### Statistical considerations and analysis

The primary end points were a comparison of disease control and treatment-related side effects. The trial was originally designed to have 80% power to detect an improvement in biochemical (PSA) control of 14% (50% increasing to 64%) and in local tumour control of 10% (80% increasing to 90%) 5 years after treatment (*α*=0.05, one sided). It was calculated that 314 men would be required. Similar numbers were needed to exclude a 10% (15% rising to 25%) increase in Grade 2 (clinically relevant) late side effects (*α*=0.05, one sided). Allowing for 10% of the study population to be unevaluable, 350 men were required for trial completion. However, as described above, recruitment was stopped after 127 men had been randomised in favour of the national protocol. Results presented here therefore are primarily hypothesis generating.

The treatments were evaluated in terms of biochemical control (PSA failure) and acute and late toxicities. Time to PSA failure was calculated from the date of randomisation with patients censored at the date of the last follow-up or death. Cumulative survival curves were constructed as time to event plots by the Kaplan–Meier method. Differences between the curves were tested for significance using the logrank test. Cox regression models were used to calculate treatment effects on time to PSA failure; these are presented as hazard ratios (HR) with their 95% confidence limits. Prostate-specific antigen nadir levels were compared using the Mann–Whitney *U*-test. Differences in acute toxicities and late toxicities reported using the RTOG criteria were analysed using the *χ*^2^ test for trend using groups based on grade 0/1/2+ and 0/1/2/3+, respectively. Differences in late radiation effects reported using the LENT SOM questionnaire were analysed using the *χ*^2^ test (no symptoms *vs* any symptoms). Although the use of one-sided tests may be appropriate as increases in both dose and treatment volume could only be associated with higher rates of disease control and complications, to avoid any chance of misrepresenting our findings two-sided significance levels are reported throughout. Statistical analyses were performed using SPSS (version 11.5.1) and STATA (version 7.0) software packages.

## RESULTS

**Figure 1:**
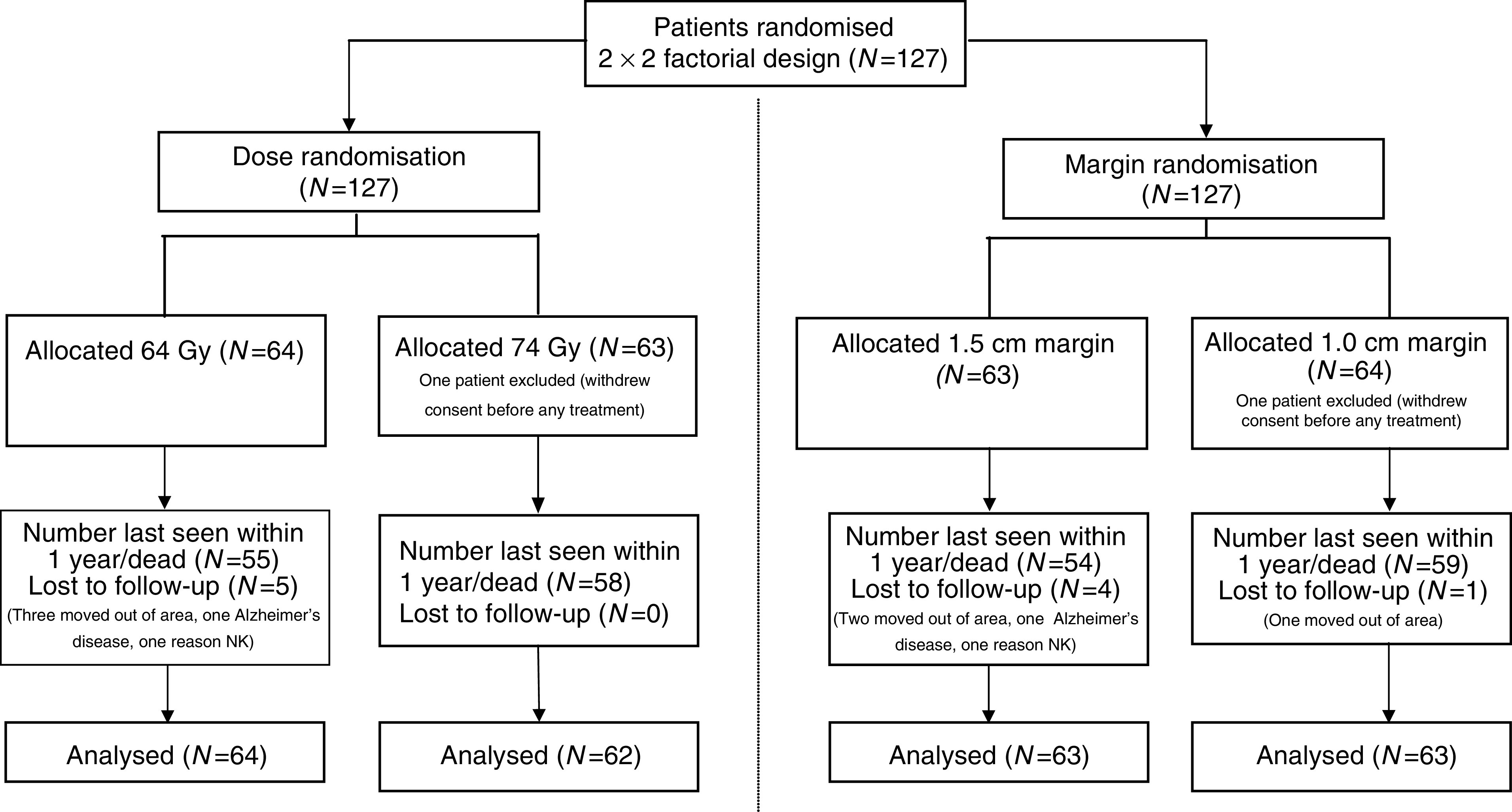
Consort diagram.

Between July 1995 and December 1997, 127 men were randomised. One patient withdrew before any treatment was given and is excluded from this analysis (Figure 1). All other men received their allocated treatments. The median age of patients was 67 years (interquartile range (IQR) 62–72 years). The median presenting PSA level was 14 ng ml^−1^ (range 1–142 ng ml^−1^). Of the 126 men, 21% had T1b/T1c cancers, 50% T2 cancers and 29% clinical T3 cancers. Histologically 18% of cancers were well differentiated or had Gleason Scores of 2–4, 72% were moderately differentiated or had Gleason Scores of 5–7 and 10% were poorly differentiated or had Gleason Scores of 8–10. Presenting features were in general well balanced between the randomised groups (Table 2). Although there were more T3 cancers in the 64 Gy dose group (*P*=0.03), this was balanced by more patients with poorly differentiated cancers in the 74 Gy dose group so that the calculated risk of seminal vesicle involvement (the stratification parameter) was similar between the groups with overall 29% of men having a low risk and 71% a moderate risk of seminal vesicle involvement. Phase I (unshaped) treatment field areas were similar in the 64 and 74 Gy groups, 101 cm^2^ (range 62–151 cm^2^) and 96 cm^2^ (range 52–146 cm^2^) for the anterior fields and 98 cm^2^ (range 52–154 cm^2^) and 93 cm^2^ (range 53–136 cm^2^) for the lateral fields, respectively. As expected, the treatment field areas were larger in the 1.5 cm margin than 1.0 cm margin groups; 111 cm^2^ (range 52–151 cm^2^) and 94 cm^2^ (range 57–114 cm^2^) for the anterior fields and 110 cm^2^ (range 69–154 cm^2^) and 86 cm^2^ (range 52–118 cm^2^) for the lateral fields, respectively. The median size of the lateral boost field (unshaped) in the 74Gy group was 31 cm^2^ (range 23–55 cm^2^). The median follow-up of all patients is 6.2 years (range 0.6–8.2 years) and 98 out of 105 (93%) of living patients have at least 5 years follow-up.

### Tumour control

Of the 126 men, 56 (44%) have developed biochemical PSA failure. Five men have developed clinically detectable local failure, and 15 have developed metastatic disease (bone 13, nodal three, lung two, liver two). A total of 21 patients have died, nine from prostate cancer and 12 from other causes. In total, 27 men have recommenced hormonal treatment for recurrent disease. All clinically detectable failures were preceded by biochemical failure (lead time, 10.7 months; IQR, 2.6–20.0 months). Of the 56 men with biochemical failure, 30 remain on an observation policy without further treatment. We recommended restarting hormonal therapy if there was clinical, radiological or bone scan evidence of metastatic disease or a rapid PSA doubling time (⩽6 months); additionally, some patients preferred immediate to deferred hormonal therapy for PSA only failure.

**Figure 2:**
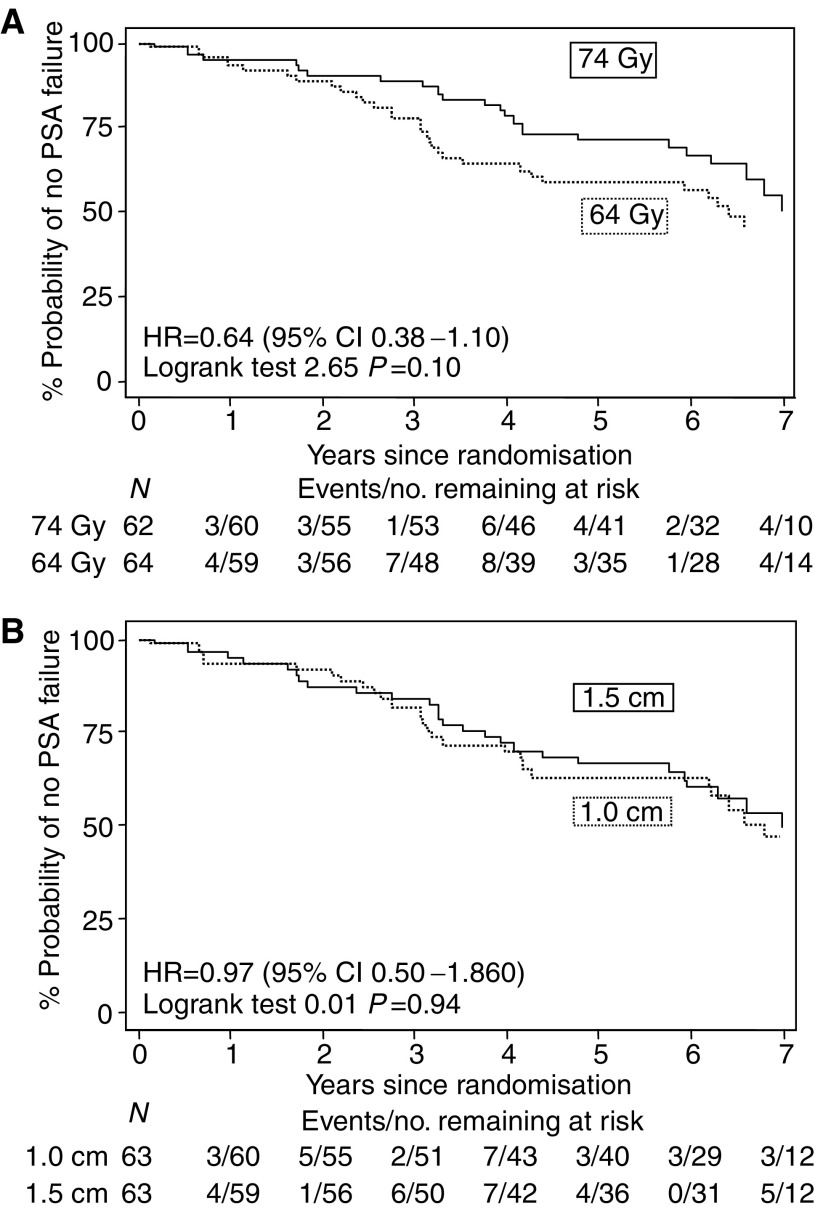
(**A**) Time to PSA failure: 74 *vs* 64 Gy dose randomization; (**B**) Time to PSA failure: 1.5 *vs* 1.0 cm margin randomization.

Comparing the randomised groups, 33 PSA failures have occurred in the standard dose (64 Gy) group compared to 23 in the escalated dose group (74 Gy) (logrank test *P*=0.10: hazard ratio (HR) 0.64, 95% CI 0.38–1.10) (Figure 2A). No difference in outcome has been seen in the margin randomisation comparison (28 events in each group, hazard ratio 0.97, 95% CI 0.50–1.86 *P*=0.94) (Figure 2B). The 5-year actuarial control rates are 59% (95% CI 45–70%) and 71% (95% CI 58–81%) in the 64 and 74 Gy groups and 67% (95% CI 53–77%) and 63% (95% CI 50–74%) for the 1.0 and 1.5 cm margin groups, respectively (Table 3).

There is a suggestion that PSA nadir levels in the 6–24 months after radiotherapy are lower in the 74 Gy group than the 64 Gy group with median levels of 0.3 ng ml^−1^ (IQR 0.1–0.5 ng ml^−1^) compared with 0.5 ng ml^−1^ (IQR 0.2–0.8 ng ml^−1^) for the 64 Gy group (*P*=0.003). Nadir levels for the 1.0 and 1.5 cm margin groups were similar with median levels of 0.4 ng ml^−1^ (IQR 0.1–0.7) and 0.3 ng ml^−1^ (IQR 0.1–0.6 ng ml^−1^) (*P*=0.45).

Of the 19 patients with local or metastatic failure, 12/7 were from the 64/74 Gy and 11/8 from the 1.0/1.5 cm margin groups, respectively. Hormonal therapy was restarted in 16/11 men from the 64/74 Gy groups, respectively. There was no difference in the time to restarting hormone therapy between the randomised groups (5-year actuarial rate 15–16% for each group). Seven of the nine prostate cancer deaths were in men treated in the 64 Gy group.

### Acute side effects of treatment

**Figure 3:**
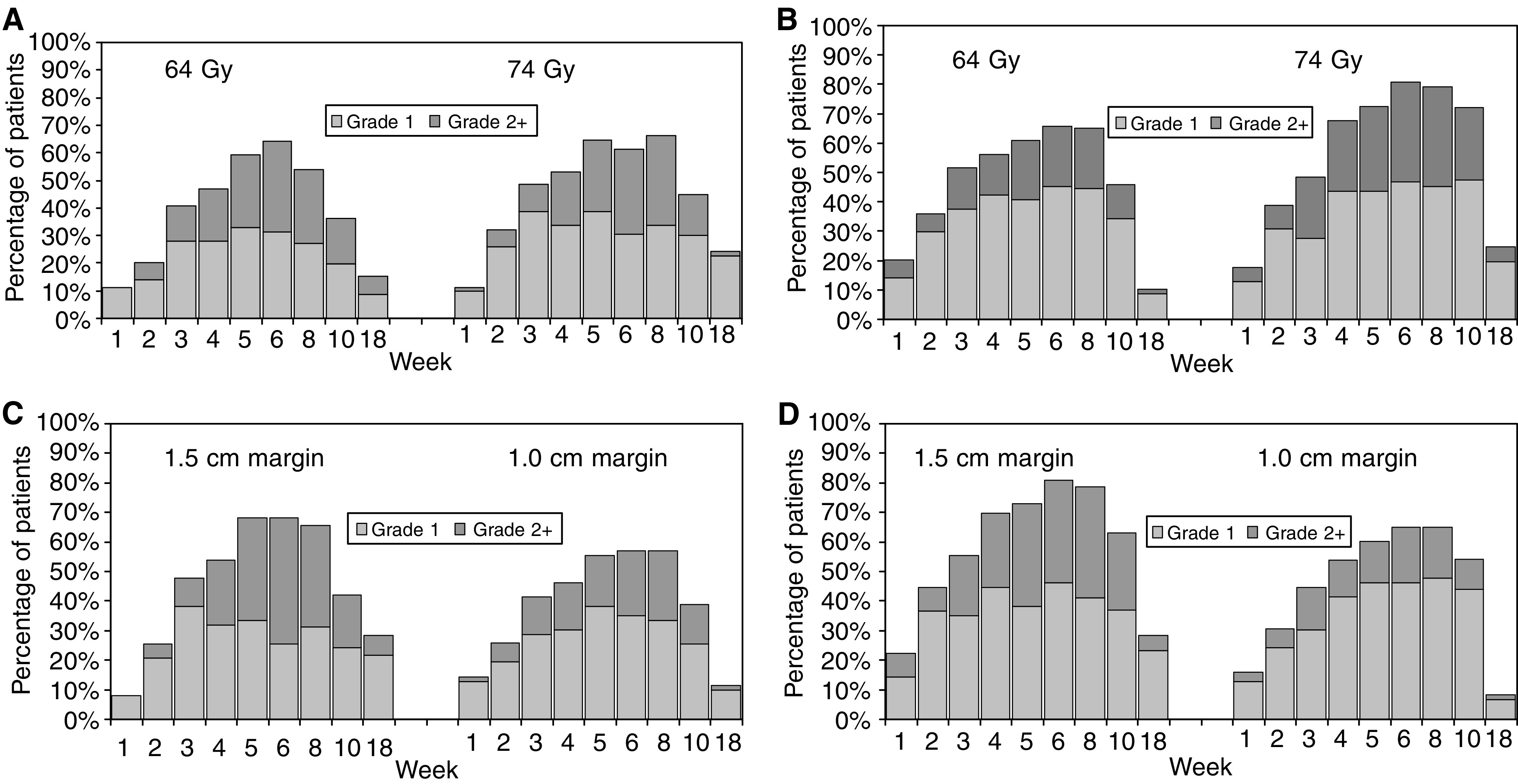
Acute bowel and bladder toxicities by dose and margin randomization. (**A**) Acute bowel toxicity (RTOG) by dose randomization; (**B**) Acute bladder toxicity (RTOG) by dose randomization; (**C**) Acute bowel toxicity (RTOG) by margin randomization; (**D**) Acute bladder toxicity (RTOG) by margin randomization.

Men were assessed at 10 time points (preradiotherapy to 18 weeks postradiotherapy). In all, 1252 out of 1260 (99%) of assessments were performed and have data available for analysis. In general, treatment was well tolerated and 69 men (55%) and 73 men (58%) had, at worst, bowel or bladder toxicity of Grade 1, at any time during weeks 1–18. Overall, three patients had Grade 3 bowel toxicity and 15 Grade ⩾3 urinary toxicity – most commonly frequency at hourly intervals or greater (Grade 3), with two men requiring catheterisation for urinary obstruction (Grade 4). Table 4 shows acute RTOG toxicity scores reported during treatment (weeks 1–6) and after treatment (weeks 8–18) by the randomised group. The time course of the ‘ wave’ of acute reaction is shown in Figure 3A–D. It would have been unexpected to see differences between the 64 Gy/74 Gy dose groups during treatment as the initial 32 daily treatments were identical but if ‘volume’ effects exist for acute bowel/bladder radiation reactions, these might be seen either during or after treatment. Concerning the dose randomization, no significant differences were seen in bowel or bladder reactions during radiotherapy (weeks 1–6), or for bowel reaction after treatment. However, acute bladder toxicity was more marked after treatment in the 74 Gy group (*P*=0.006): at 10 weeks, toxicity scores were still elevated although in the 64 Gy group symptoms were beginning to settle. Side effects substantially improved by week 18, and only four men had residual greater than Grade 1 symptoms remaining in either group (Figure 3A). The total duration of treatment (7.5 *vs* 6.5 weeks) as well as total dose could be related to the differences observed. Concerning the margin randomization, the 1.0 cm margin group had less bowel toxicity during radiotherapy (*P*=0.05) and these differences were most obvious during weeks 5 and 6 of treatment (Figure 3C). Although there was no difference in the maximum toxicity after treatment, which in general settled rapidly, less men had residual bowel problems (RTOG Grade ⩾1) in the 1.0 cm margin group at week 18 (11% 1.0 cm margin, 28% 1.5 cm margin, *P*=0.02) and only five of the 126 men remained with Grade >1 toxicity. Bladder toxicity was more marked both during and after treatment in the 1.5 cm margin group (*P*=0.002, *P*=0.02, respectively) as seen in Table 4 and Figure 3D. Again, as with the bowel reactions, differences during treatment were seen most obviously during weeks 5 and 6 and toxicity settled more quickly in the 1.0 cm margin group so that 92% of men were free of treatment-related symptoms by week 18 compared to 72% in the 1.5 cm margin group (*P*=0.004). However, only four out of 126 men remained with Grade >1 side effects.

### Late side effects of treatment

**Figure 4:**
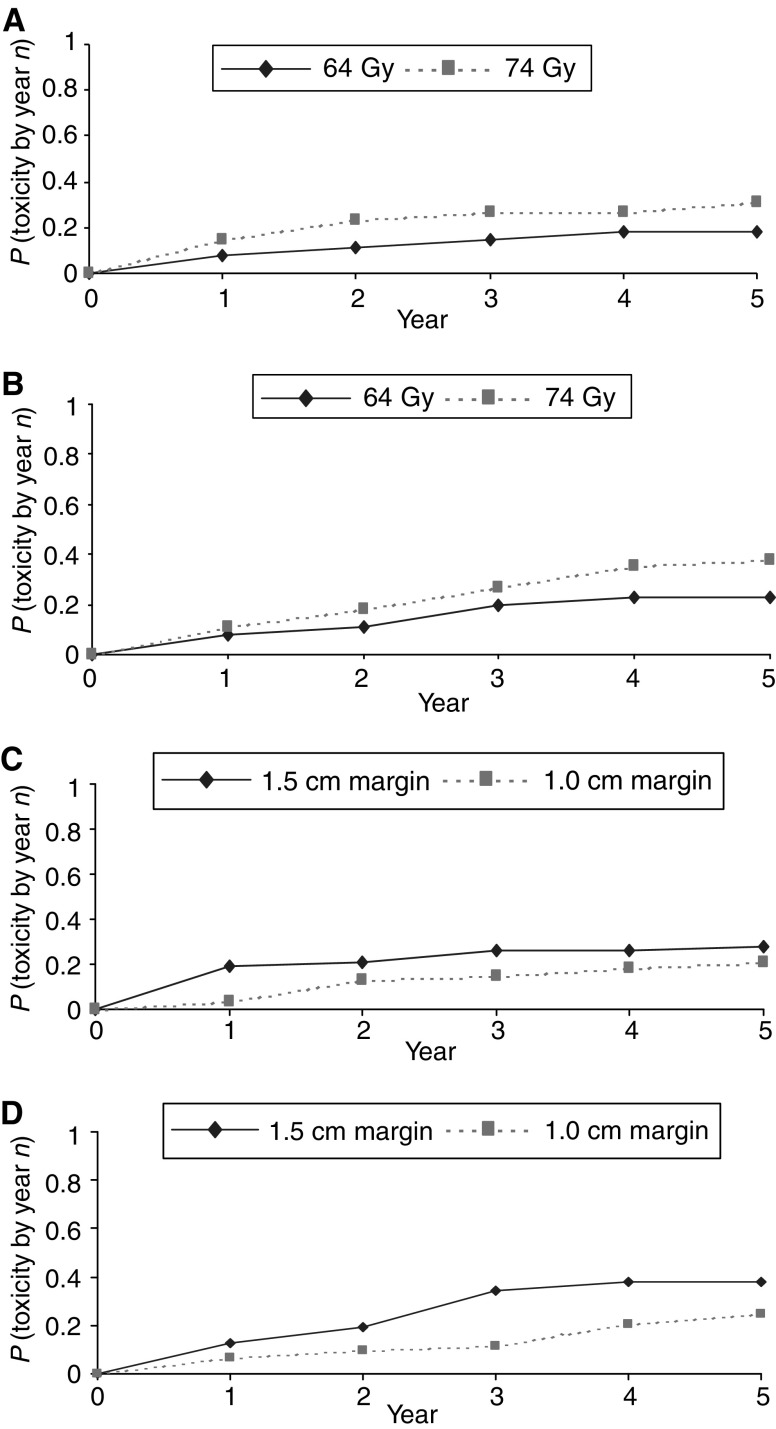
Cumulative grade ⩾2 bowel and bladder toxicities by dose and margin randomization. (**A**) Cumulative bowel toxicity grade ⩾2 by dose; (**B**) cumulative bladder toxicity grade ⩾2 by dose; (**C**) cumulative bowel toxicity grade ⩾2 by margin; (**D**) cumulative bladder toxicity grade ⩾2 by margin.

Late side effects were assessed using standard RTOG and LENT SOM physician completed questionnaires. Annual RTOG assessments 1–5 years after radiotherapy were available in 123, 118, 113, 102 and 92 patients and LENT SOM data for 115, 111, 94, 94 and 90 men, respectively. Table 5 shows the cumulative incidence of late side effects at 2 years. Overall, 83/85% of men had at most Grade 1 bowel/bladder toxicities reported. After 2 years of follow-up, RTOG grade ⩾2 side effects were seen as follows: rectal bleeding 16, rectal discomfort three, bowel frequency three, and day time urinary frequency 10, nocturia seven, dysuria one, haematuria one, urinary incontinence three and urethral dilatation two. Comparing the randomised groups (Table 5), Grade ⩾2 bowel complications were more common in the 74 Gy and 1.5 cm margin groups (*P*=0.02, and *P*=0.005, respectively) and, of note, all three Grade 3 complications occurred in men treated to 74 Gy and with a 1.5 cm margin. No significant differences were seen in bladder toxicity although there is a suggestion of more Grade ⩾2 complications in the 74 Gy and 1.5 cm margin groups (*P*=0.28 and *P*=0.30, respectively). Actuarial projections of the cumulative incidence of Grade ⩾2 bowel and bladder complications are shown in Figure 4A–D. A persisting trend for higher complication rates in the 74 Gy and 1.5 cm margin groups is seen.

Baseline LENT SOM assessment showed that 14 out of 88 (16%) men had some rectal dysfunction. In 13 of the 14, there was occasional rectal bleeding or bowel frequency 2–4 times per day. This increased to 35–44% during years 1–5 of follow-up (Table 6), but any Grade 3 toxicity was uncommon being recorded in 8% or less of men on any particular follow-up visit. Of the 41 Grade 3 rectal toxicity scores reported in 32 men, 19 were due to rectal bleeding, six ulceration, three tenesmus, two mucosal loss, two stool frequency and one pain. The pattern of toxicity changed with time, increased bowel frequency and tenesmus was recorded most commonly at 6 months and 1-year follow-up. Thereafter, approximately two-thirds of toxicity was due to rectal bleeding, the remainder being caused by a constellation of tenesmus, mucosal loss and sphincter disturbance. Comparison between the treatment groups suggested an increased complication rate in the dose escalated (74 Gy) dose group, but this reached statistical significance (*P*=0.02) at the 4-year follow-up point only. However, the 1.5 cm margin group had a consistently higher complication rate after treatment. The toxicity grade using RTOG and LENT SOM systems cannot be exactly ‘translated’ but the results using the two different systems appeared similar.

Using the LENT SOM classification, 42% of men had bladder symptoms at baseline; frequency and poor urinary stream were scored in all of these 37 men. At 6 months, only 27% of men were symptomatic and this level remained constant during the remainder of follow-up. Although consistently higher symptom scores were recorded in the 1.5 cm margin group, this reached statistical significance only at 3 years (Table 6). At 6 months, there was a statistically significant increase in urinary frequency in the 74 Gy compared with 64 Gy group with Grade ⩾3 scores seen in seven out of 39 (18%) and three out of 43 (7%) patients, respectively (*P*=0.03). This is consistent with the higher acute toxicity seen at week 18 in the 74 Gy group (Figure 2B). Overall, urinary symptoms remained higher after 1 year in the 74 Gy group (*P*=0.02), but this reduced on longer term follow-up (Table 6). These differences were not detected using the RTOG scoring system. At 6 months, 26 out of 37 (70%) of symptoms scored, and at 12 months, 35 out of 61 (57%) of symptoms scored were of Grade ⩾2 urinary frequency (frequency at 2–3-h intervals or less), intermittent poor stream and dysuria on the LENT SOM system which may not have registered on the RTOG grading system.

The LENT SOM classification of sexual functioning comprises questions related to adequacy of erectile function, dryness, desire, satisfaction, frequency of intercourse and orgasm and management of symptoms. At baseline, 38% of men reported some sexual dysfunction (Grade ⩾1), this increased to 66% at 6 months and 1 year and 81% by year 5. Some Grade ⩾3 sexual dysfunction was reported by 26% of men at baseline, which increased to 52% at 6 months, 61% at 1 year and thereafter remained stable being 57% at 5 years. Between 13 and 21% of men used some form of treatment for sexual dysfunction at assessments between 2 and 5 years after treatment. Comparing the randomised treatment groups at each time point, no differences were seen except for erectile function (Grade ⩾3 63/42%, *P*=0.04) and orgasm (Grade ⩾3 59/38%, *P*=0.04) for 74/64 Gy groups, respectively, 2 years after treatment. As multiple comparisons were made, these findings must be treated with caution.

No ureteric side effects were recorded and bone/skin symptoms (⩽2% at any assessment) were similar to those at baseline.

## DISCUSSION

The results of this randomised pilot study suggest that dose escalation improves biochemical (PSA) control of disease and that both radiation dose and technique impact on radiation-related side effects. The actuarial 5-year failure-free estimate increased from 59% (95% CI: 45–70%) to 71% (95% CI: 58–81%), comparing the 64 and 74 Gy treatment groups. These results are quite similar to the findings of the only other reported phase III randomised trial that was performed at the MD Anderson (Pollack *et al*, 2000). In this trial, which included 305 men, the total radiotherapy dose was randomised to either 70 or 78 Gy but no initial hormone treatment was given. The 5-year PSA control rates were 69 and 79%, respectively (*P*=0.06). This trial included patients with better prognostic features (for example, median PSA 8 ng ml^−1^ compared to 14 ng ml^−1^ in our current study) (Shipley *et al*, 1999; Parker *et al*, 2002). Subgroup analysis of the 106 men with presenting PSA levels greater than 10 ng ml^−1^ showed biochemical control rates of 48 and 75% (*P*=0.01) for the 70 and 78 Gy groups, respectively. Improvements in PSA control rates of similar magnitude have also been reported from phase II studies in larger groups of men (Sandler *et al*, 1992; Hanks *et al*, 1998; Zelefsky *et al*, 1998; Fiveash *et al*, 2000; Kupelian *et al*, 2000). For example, the Memorial Sloan Kettering Group have reported outcome from 1100 men comparing doses in the range of 64–70 Gy and 76–86 Gy (Zelefsky *et al*, 2001). Using clinical stage, histological grade and presenting PSA to define prognostic groups showed 5-year actuarial PSA control rates in 77 *vs* 90% (*P*=0.05), 50 *vs* 70% (*P*=0.001) and 21 *vs* 47% (*P*=0.002) of favourable, intermediate and unfavourable risk cases treated to lower or higher doses, respectively. A critical issue is whether or not PSA control will clearly relate to disease recurrence or to overall survival. A retrospective analysis from the RTOG suggests that dose escalation may indeed be related to improved survival. In their study, which included 1465 men treated in four protocols between 1975 and 1992, men with high-grade cancers who received higher radiation doses (⩾66 *vs* <66 Gy) had a 20% lower risk of death from prostate cancer and a 27% reduction in overall mortality. This benefit was not seen in men with well- or moderately differentiated cancers (Valicenti *et al*, 2000). However, these retrospective studies of sequentially treated cohorts of patients may be subject to bias from ‘stage migration’, which has occurred during the 1990s, resulting in an apparent overall improvement in treatment outcome (D'Amico *et al*, 2002). Prospective randomised trials are therefore needed and studies that are being undertaken in the UK (MRC RT01 Trial), The Netherlands, France and North America will recruit, in total, over 3000 men. When available, these trials should clarify the benefit of dose escalation in men with disease in different prognostic subgroups.

Our analysis of acute treatment-related side effects shows that, in general, treatment was well tolerated but that short lasting increases in bladder and bowel toxicity were seen in the 1.5 cm margin group and an increase in bladder, but not bowel, side effects was seen shortly after completion of radiotherapy in the 74 Gy group. Although such differences were measurable and statistically significant, they were arguably of little clinical significance as side effects settled within 3 months of treatment completion. The incidence of late rectal toxicity varies considerably in other studies, Grade 2 or more side effects being reported in 2–32% of patients (Lee *et al*, 1996; Schultheiss *et al*, 1997; Hanks *et al*, 1998; Zelefsky *et al*, 1999, 2001; Bey *et al*, 2000; Michalski *et al*, 2000; Skwarchuk *et al*, 2000). Risk factors include dose, technique, past history of diabetes mellitus, rectal volume and the occurrence of acute toxicity (Schultheiss *et al*, 1997; Denham *et al*, 1999; Skwarchuk *et al*, 2000). In this study, late side effects following radiotherapy were in line with our previous experience (Dearnaley *et al*, 1999), and 2 years after therapy, 17% of men had experienced Grade 2 or more bowel toxicity and 15% Grade 2 or more bladder side effects. At 2 years after radiotherapy, there was a statistically significant increase in bowel toxicity for both the 74 Gy and 1.5 cm margin groups. Although an excess of bladder side effects was seen at this time point, they did not reach statistical significance; however, on further follow-up, the differences in bladder toxicity became more obvious and statistically significant for the margin randomisation. In general, the LENT SOM assessments gave similar findings to the RTOG assessments as reported by other groups (Anacak *et al*, 2001), although the LENT SOM assessment was more sensitive at picking up minor degrees of bladder impairment, which were seen in both the 74 Gy and 1.5 cm margin groups. It is noteworthy that after treatment, the degree of bladder dysfunction was decreased from pretreatment assessments, probably reflecting the improvement in lower urinary tract symptoms as a result of prostate gland shrinkage.

Our current study is the only randomised trial in prostate cancer to address the issue of radiation ‘safety margin’ after initial hormone therapy. Two separate issues arise. Firstly, is it the prehormone or posthormone prostate volume that should be used to define the radiation clinical target volume. Secondly, what is the appropriate margin to account for geometric uncertainties in treatment planning and delivery as well as prostate movement. The choice of a 1.0 or 1.5 cm margin in this trial was designed to address the former question but clearly the improvements in treatment accuracy that have occurred as treatment verification techniques have become more sophisticated using, for example, electronic portal imaging (Mubata *et al*, 1998) could have influenced our results. However, we found no evidence for a detriment in PSA control using a 1.0 cm margin to the posthormone treatment target volume. Despite the necessarily wide confidence limits on this result from a small study, we could not justify continuing to use a 1.5 cm margin, as this larger treatment volume was clearly related to an increase in radiation-related side effects. This information was considered by the MRC RT01 Data Monitoring Committee and it was agreed that Trial Centres should be advised to use the smaller 1.0 cm margin (in the RT01 trial, the margin randomisation was omitted and margins of 1.0–1.5 cm were chosen by the treating clinician). Several groups are developing models based on dose volume histograms to predict the likelihood of complications (Lu *et al*, 1995; Boersma *et al*, 1998; Fiorino *et al*, 2003; Jackson *et al*, 2001; Wachter *et al*, 2001). This study will add a very useful further patient data set to complement our previous studies (Fenwick *et al*, 2001), which will be combined with further information from the MRC RT01 trial. It is already clear, however, that careful attempts to shield the rectum using precise radiotherapy delivery methods can result in very low toxicity profiles (Lee *et al*, 1996; Zelefsky *et al*, 1999). Increasing standards of treatment verification and accuracy (Amer *et al*, 2001) together with studies using marker seeds (Crook *et al*, 1995; Wu *et al*, 2001) or dynamic magnetic resonance imaging (Padhani *et al*, 1999) have clarified the extent of prostate movement. This is dependent on rectal distension and for patients with an empty rectum on CT planning scans, more restricted posterior margins may well be adequate and would lead to a further decrease in the volume of the rectum treated with an expected further reduction in treatment-related side effects.

In this trial, we used initial androgen suppression in conjunction with conformal dose escalated radiotherapy as they are complementary treatment approaches in that both achieve improvement in local treatment control and reduce radiation treatment volumes (Dearnaley, 2001). Although androgen levels return to normal in over 90% of men after short-course hormone therapy (Shahidi *et al*, 2001), sexual dysfunction may be more common after combined modality treatment. Future results from ongoing phase III trials will help to define optimal contributions from high-dose conformal radiation or combined modality treatments with the aim of balancing the relative effectiveness and toxicities of these different treatment approaches. Newer antiandrogens such as Bicalutamide will need to be included in future treatment strategies. For more advanced cancers, there is now good evidence that prolonged courses of adjuvant hormonal therapy are of additional benefit (Roach III *et al*, 2000; Horwitz *et al*, 2001; Bolla *et al*, 2002; Pilepich *et al*, 2003), but it is not yet known whether dose escalation will produce further advantage.

The MRC RT01 trial completed recruitment of over 850 patients in December 2001. The remaining ongoing dose escalation trials (see above) give doses of 68–73 Gy in the control groups and 78–82 Gy in the escalated dose groups. It is now a priority for the radiotherapy community to deliver such treatments safely. A further challenge has come from the hypothesis that the *α*–*β* ratio for prostate cancer may be low, which implies that there would be a therapeutic advantage from treating with large doses per fraction in hypofractionated schedules (Duchesne and Peters, 1999; Brenner, 2000; King and Fowler, 2001). Such schedules, if effective and safe, would be more convenient for patients and make better use of sophisticated resources. Appropriate trials to address these questions are currently under way in the UK and elsewhere.

## Figures and Tables

**Table 1:** Definition of radiation target volume

**Radiation dose**	**Risk group^a^**	**Gross tumour volume (GTV)**	**Clinical target volume (CTV)**	**Planning target volume (PTV)**
*Phase I*				
64 and 74 Gy groups	Low-risk SV involvement^b^	Prostate+base SV	GTV1+0.5 or 1.0 cm^c^	CTV1+0.5 cm
	Moderate-risk SV involvement^d^	Prostate+SV	GTV1+0.5 or 1.0 cm^c^	CTV1+0.5 cm
				
*Phase II*				
74 Gy group	All cancers	Prostate only	GTV2 (no added margin)	CTV2 (no added margin)

a Risk of seminal vesicles (SV) involvement (%)=prostate-specific antigen (PSA) +(Gleason score−6 × 10) for T1/T2 cancers (Roach III, 1993).

b T1/T2 cancers with SV involvement risk <15%.

c Randomised treatment options.

d T3/T4 cancers and T1/T2 cancers with SV involvement risk ⩾15%.

**Table 2:** Patient and tumour characteristics

	**64 Gy dose**	**74 Gy dose**	**1.0 cm margin**	**1.5 cm margin**	**Total**
No.	64	62	63	63	126
Age (years): median (IQR)	66 (62–71)	69 (63–72)	66 (62–72)	68 (63–72)	67 (62–72)
PSA (ng ml^−1^) at baseline, median (IQR)	15 (7–28)	14 (7–29)	13 (7–31)	15 (7–26)	14 (7–29)
					
Clinical stage* *(%)*					
T1	17	26	17	25	21
T2	44	56	52	48	50
T3	39	18	30	27	29
					
*Histology (%)*					
Grade 1	19	18	14	22	18
Grade 2	77	68	79	65	72
Grade 3	5	15	6	13	10
					
*Risk of seminal vesicle involvement (%)*					
Low	31	27	30	30	29
Moderate	69	73	70	70	71

* *P*=0.03, *χ*^2^ test 64 *vs* 74 Gy. PSA=prostate-specific antigen; IQR=interquartile range.

**Table 3:** Time to prostate-specific antigen (PSA) failure analysis

**% PSA failure free (95% CI)**				
Year	64 Gy dose	74 Gy dose	1.0 cm margin	1.5 cm margin
2	89 (78–95)	90 (80–95)	87 (76–93)	92 (82–97)
3	78 (65–86)	89 (77–94)	84 (72–91)	82 (70–90)
4	64 (51–75)	78 (65–87)	72 (59–82)	70 (57–80)
**5**	**59 (45–70)**	**71 (58–81)**	**67 (53–77)**	**63 (50–74)**
6	57 (43–68)	67 (53–78)	60 (53–77)	63 (50–74)

Bold represents 5-year results mentioned in text.

**Table 4:** Acute bowel and bladder toxicity (RTOG scoring): by dose and margin randomisations

	**64 Gy dose (*N*=64)**		**74 Gy dose (*N*=62)**		**1.0 cm margin (*N*=63)**		**1.5 cm margin (*N*=63)**		**Total (*N*=126)**	
	***n***	**(%)**	***n***	**(%)**	***n***	**(%)**	***n***	**(%)**	***n***	**(%)**
*Weeks 1–18*										
*Maximum bowel toxicity*										
0	14	(22)	10	(16)	13	(21)	11	(17)	24	(19)
1	22	(34)	23	(37)	27	(43)	18	(29)	45	(36)
2	25	(39)	29	(47)	22	(35)	32	(51)	54	(43)
3	3	(5)	0	(0)	1	(2)	2	(3)	3	(2)
4	0	(0)	0	(0)	0	(0)	0	(0)	0	(0)
	(*P*=0.52)				(*P*=0.13)					
*Maximum bladder toxicity*										
0	15	(23)	8	(13)	14	(22)	9	(14)	23	(18)
1	26	(41)	24	(39)	33	(52)	17	(27)	50	(40)
2	16	(25)	22	(35)	10	(16)	28	(44)	38	(30)
3	7	(11)	6	(10)	5	(8)	8	(13)	13	(10)
4	0	(0)	2	(3)	1	(2)	1	(2)	2	(2)
	(*P*=0.08)				(*P*=0.002)					

*Weeks 1–6*										
*Maximum bowel toxicity*										
0	17	(27)	16	(26)	20	(32)	13	(21)	33	(26)
1	22	(34)	20	(32)	23	(37)	19	(30)	42	(33)
2	24	(38)	26	(42)	20	(32)	30	(48)	50	(40)
3	1	(2)	0	(0)	0	(0)	1	(2)	1	(1)
4	0	(0)	0	(0)	0	(0)	0	(0)	0	(0)
	(*P*=0.8)				(*P*=0.05)					
*Maximum bladder toxicity*										
0	18	(28)	12	(19)	20	(32)	10	(16)	30	(24)
1	26	(41)	25	(40)	29	(46)	22	(35)	51	(40)
2	13	(20)	17	(27)	8	(13)	22	(35)	30	(24)
3	7	(11)	6	(10)	5	(8)	8	(13)	13	(10)
4	0	(0)	2	(3)	1	(2)	1	(2)	2	(2)
	(*P*=0.19)				(*P*=0.002)					

*Weeks 8–18*										
*Maximum bowel toxicity*										
0	28	(44)	17	(27)	25	(40)	20	(32)	45	(36)
1	16	(25)	23	(37)	21	(33)	18	(29)	39	(31)
2	18	(28)	22	(35)	16	(25)	24	(38)	40	(32)
3	2	(3)	0	(0)	1	(2)	1	(2)	2	(2)
4	0	(0)	0	(0)	0	(0)	0	(0)	0	(0)
	(*P*=0.16)				(*P*=0.16)					
*Maximum bladder toxicity*										
0	22	(34)	9	(15)	17	(27)	14	(22)	31	(25)
1	27	(42)	28	(45)	34	(54)	21	(33)	55	(44)
2	10	(16)	19	(31)	9	(14)	20	(32)	29	(23)
3	5	(8)	4	(6)	2	(3)	7	(11)	9	(7)
4	0	(0)	2	(3)	1	(2)	1	(2)	2	(2)
	(*P*=0.006)				(*P*=0.02)					

Trend tests (*χ*^2^_1_) based on toxicity level grouping: 0, 1, ⩾2.

**Table 5:** Late bladder and bowel toxicity (RTOG scoring) by dose and margin randomisations. Maximum toxicity in first 2 years

	**No. (%) of men with toxicity score**				
	**64 Gy dose (*N*=63)**	**74 Gy dose (*N*=61)**	**1.0 cm margin (*N*=62)**	**1.5 cm margin (*N*=62)**	**All Patients (*N*=124)**
*Bowel toxicity*					
Grade 0	30 (48)	20 (39)	33 (53)	17 (27)	50 (40)
1	26 (41)	27 (44)	21 (34)	32 (52)	53 (43)
2	7 (11)	11 (18)	8 (13)	10 (16)	18 (15)
3	0	3 (5)	0	3 (5)	3 (2)
4	0	0	0	0	0
	*P*=0.02		*P*=0.005		
					
*Bladder toxicity*					
Grade 0	33 (52)	24 (39)	29 (47)	28 (45)	57 (46)
1	23 (37)	26 (43)	27 (44)	22 (35)	49 (40)
2	5 (8)	8 (13)	5 (8)	8 (13)	13 (10)
3	2 (3)	3 (5)	1 (2)	4 (6)	5 (4)
4	0	0	0	0	0
	*P*=0.17		*P*=0.27		

**Table 6:** Late radiation side effects on rectum and bladder: LENT SOM grading system

			**No. (%) of men with symptoms**				
	**Time of measurement**	**No. assessed**	**64 Gy dose**	**74 Gy dose**	**1.0 cm margin**	**1.5 cm margin**	**All patients**
Any bladder symptoms	Pretreatment	88	22 (45)	15 (38)	16 (35)	21 (50)	37 (42)
(Grades 1–4)	6 months	82	7 (16)*	15 (38)*	8 (19)	14 (36)	22 (27)
	1 year	115	11 (19)*	22 (39)*	12 (21)	21 (36)	33 (29)
	2 years	111	10 (18)	17 (30)	11 (20)	16 (29)	27 (24)
	3 years	94	12 (26)	10 (21)	5 (12)****	17 (33)****	22 (23)
	4 years	94	11 (23)	14 (30)	10 (22)	15 (31)	25 (27)
	5 years	90	11 (24)	17 (38)	13 (30)	15 (33)	28 (31)
							
Any rectal symptoms	Pretreatment	88	8 (16)	6 (15)	7 (15)	7 (17)	14 (16)
(Grades 1–4)	6 months	82	10 (23)	13 (33)	13 (30)	10 (26)	23 (28)
	1 year	115	16 (28)	24 (42)	14 (25)*	26 (45)*	40 (35)
	2 years	111	18 (38)	28 (50)	18 (32)**	28 (51)**	46 (40)
	3 years	94	14 (30)	22 (47)	10 (23)***	26 (51)***	36 (38)
	4 years	94	13 (27)*	23 (50)*	14 (31)	22 (45)	36 (38)
	5 years	90	17 (38)	23 (51)	17 (39)	23 (50)	40 (44)

Significance levels comparing randomised groups: 64 *vs* 74 Gy or 1.0 cm margin *vs* 1.5 cm margin.

* *P*=0.02;

** *P*=0.05;

*** *P*=0.005;

**** *P*=0.013.

Pretreatment in this table signifies before *any* treatment has begun, that is, before both hormones and RT.
